# Factors Contributing to Nurses' Intention to Leave the Profession: A Qualitative Study in Catalonia, Spain, following the Latest Waves of COVID-19

**DOI:** 10.1155/2024/7971020

**Published:** 2024-05-30

**Authors:** Carolina E. Watson, Maria Dolors Bernabeu-Tamayo, David Giménez-Díez, Manuel Lillo-Crespo, Juan M. Leyva-Moral

**Affiliations:** ^1^Autonomous University of Barcelona, GRIVIS Research Group, Nursing Department, Faculty of Medicine, Av. Can Domènech s/n, Bellaterra 08193, Spain; ^2^HLA Vistahermosa Hospital, Alicante, Spain; ^3^Nursing Department, University of Alicante, Alicante, Spain

## Abstract

**Introduction:**

The COVID-19 pandemic has had a significant impact on healthcare professionals globally, with nurses facing diverse challenges at the forefront. Despite their resilience, nurses are experiencing emotional burdens, which have contributed to a growing intention to abandon the profession. Understanding these factors is crucial for addressing the global nursing shortage.

**Methods:**

A qualitative descriptive approach was utilized for this study. Nurses who were actively working during the last waves of the pandemic in Catalonia, Spain, were intentionally recruited through social media and personal contacts, and data were collected through online semistructured interviews until data saturation was reached. Data were analyzed using Braun and Clarke's thematic analysis method.

**Results:**

Fourteen nurses, with an average of 22.8 years of work experience, were interviewed. Thematic analysis revealed three main themes: (1) the impact of COVID-19 on health, (2) factors influencing the decision to stay, and (3) recommendations to improve crisis management.

**Conclusion:**

Nurses faced significant emotional impacts but demonstrated dedication and resilience. Their decision to persevere was influenced by factors such as responsibility, guilt, and economic stability. Urgent measures are necessary to provide tailored mental health support and recognize emotional challenges in crisis preparedness.

## 1. Introduction

The COVID-19 pandemic generated an unprecedented global health crisis, affecting millions of individuals worldwide, including healthcare professionals. At the forefront of this unexpected battle, nurses assumed a challenging role by caring not only for those impacted by COVID-19 but also playing an important part in preventing infection. In addition, nurses took on more responsibilities and learned new techniques and skills related to the novel situation at the start of the outbreak, including providing psychological support facing isolation, remote patient monitoring, PCR tests, among others. Despite the negative effects that nurses may have faced in terms of health and job satisfaction, they bravely dealt with the health-related challenges accompanying the pandemic, especially in the areas of mental health, isolation, and physical sequelae [1, 2]. Consequently, such ongoing struggle led to emotional and psychological burdens as well as fatigue, which impacted nurses' professional and personal well-being. This may be the reason for the continuing rise in their intention to abandon the profession, which subsequently contributes to the global nursing shortage and healthcare organizations' endeavor to provide professional, high-quality, and safe care [2].

There is considerable interest in the impact of COVID-19 on the emotional and mental well-being of healthcare providers, particularly in the initial year and early waves of the pandemic. These professionals were exposed to high levels of stress, anxiety, and physical and emotional exhaustion due to the uncertain situation they found themselves in [3]. In this context, early evidence suggests that a substantial number of healthcare workers experienced mood and sleep disturbances during the initial stages of the COVID-19 outbreak [4].

Moral distress, compassion fatigue, burnout, and post-traumatic stress disorder (PTSD) have been used to describe emotional states that lead many health professionals, including nurses, to consider abandoning the profession [5]. However, it is important to note that nurses who experience unresolved stress are more susceptible to burnout [6], which highlights the interconnectedness of these emotional challenges. Moreover, nurses working on the front lines have reported experiencing fear, anxiety, stress, social isolation, depressive symptoms, uncertainty, and frustration [7], underscoring the severity and complexity of the emotional toll faced by healthcare professionals. In line with the scientific literature, these experiences impact psychological well-being and can significantly affect job satisfaction, potentially influencing one's intention to abandon the profession [8, 9].

Burnout leads to poor job satisfaction and a higher likelihood of turnover [10]. Even prior to the COVID-19 pandemic, factors such as psychological stress, job burnout, and dissatisfaction were already related to a higher turnover intention [11]. A healthy work environment has positive effects on nurses, and nursing care is positively linked with patient outcomes [12]. This context may explain why nurses' well-being and turnover intention became serious concerns for healthcare managers and decision-makers regarding the workplace due to the additional stress experienced during the COVID-19 pandemic [8].

In some cases, during the early stages of the pandemic, nurses directly caring for COVID-19 patients, especially those who felt poorly prepared and overwhelmed, exhibited a high intention to leave their job [13]. This phenomenon of turnover intention has been explored in the literature. For instance, Yu et al. [9] suggest that during the early stages of the pandemic, the intention to leave among nurses was notably lower compared to the data provided by mass media reports, suggesting a discrepancy in perceptions. On the other hand, Raso et al. [14] indicated that 11% of a sample of over 5,000 nurses expressed an intent to leave their positions, while 20% remained undecided. However, when considering leaving the profession or being undecided about their future career path, both percentages were lower at 2% and 8%, respectively. Further insight into this phenomenon is provided by Nashwan et al. [15]; whose study conducted in Qatar during the initial waves of COVID-19 compares turnover intentions before and during the pandemic, revealing significantly higher turnover intentions during the pandemic period compared to before COVID-19.

This evidence underscores the complexity of nurses' intentions to leave their positions during the pandemic and emphasizes the importance of understanding the contextual factors that influence turnover intentions. Much of the existing literature—including studies published in 2023—relies on data collected between 2020 and 2021. It is noteworthy that during that period, the cumulative stress and professional fatigue associated with COVID-19 were still in their early stages. Consequently, these numbers may fluctuate due to prolonged exposure to the situation. The impact of the prolonged duration of the pandemic and the experiences accumulated over subsequent waves of COVID-19 in 2022 have not been as comprehensively explored in the literature to date. Hence, there is a lack of qualitative studies that specifically address this phenomenon, which are needed to deepen our understanding of healthcare professionals' experiences.

Widespread shortages of nurses on a global scale predating the pandemic [12, 16], exacerbated by the emergence of COVID-19 [17], underscore the critical need to delve into the reality experienced during the later stages of the pandemic. It is crucial to delve deeper into the underlying motivations and circumstances that have contributed to the alarmingly high rates of nurses expressing an intention to abandon their nursing practice. In this investigation, we aim to study the impact of COVID-19 on motivation and emotional well-being of Spanish nurses, specifically within the context of the sixth and seventh waves of COVID-19 (Oct 2021 to Feb 2022 and May/Jun 2022 to Aug 2022). Identifying the underlying factors that may influence nurses to consider abandoning the profession is essential, as understanding what drives nurses' intentions to leave has significant implications for the ability of health systems to cope with future health crises. Our findings may be helpful for healthcare managers, policymakers, researchers, and educators to gain a comprehensive view of this phenomenon; the results can also be utilized to design and implement strategies to help nurses cope with stress, to motivate them, to improve their well-being, and to retain them within healthcare organizations.

## 2. Methods

### 2.1. Design

We used a descriptive qualitative method to explore nurses' perspectives on their lived experiences, focusing on their intention to leave the profession alongside the factors and motivation influencing nurses' decisions to continue working as nurses despite this initial intention. Within this framework, we delve into the motivations of nurses who express the intention to leave the field, regardless of whether they have actually left or ultimately decided to stay, in order to provide a comprehensive understanding of the “intention to leave” phenomenon. This approach aims to provide clear descriptions of personal experiences and perspectives without unnecessary complexity or ambiguity [18].

### 2.2. Participants

Participants were selected through purposive sampling [19], guided by a decision-making framework aimed at achieving representativeness and diversity in terms of work experience, gender, age, and geographic location, while ensuring sample diversity among those who met the inclusion criteria. The study's inclusion criteria were to be a registered nurse who (1) had worked in the region of Catalonia, Spain, during the approximate periods of the sixth and seventh waves of the COVID-19 pandemic in this region and (2) had intention to leave the profession. This method aimed to capture a range of perspectives and experiences to enrich the analysis and interpretation. Information about the study was distributed using social media and personal contacts. Once participants contacted the principal investigator, the full details of the study were provided, and all possible doubts were dispelled in order to obtain their informed consent. Prior to each interview, participants were provided with a detailed explanation of the study and its importance, aiming to establish trust and rapport. All doubts and concerns were addressed to ensure participants felt comfortable and willing to share their experiences openly. Pseudonyms were used to ensure anonymity. The research team assured the participants that their information would be kept confidential. This was expressed openly in the informed consent form. The participants did not receive any financial incentive for their involvement. Finally, 14 nurses agreed voluntarily to participate and gave written consent to be interviewed. Even though they were informed about their freedom to withdraw from the study whenever they needed or wished to, none of them did.

## 3. Data Collection

We gathered data through semistructured interviews between January and March of 2023. Each interview was conducted within a 30–45 minute framework, and participants were given the flexibility to choose their preferred meeting format, with all opting for online convening. The interviews were carried out using Teams® by a researcher with broad experience conducting these types of interviews who did not have any direct relationship with the healthcare organizations where the nurses worked. A script was developed based on prior knowledge and research concerns, and the final version was created after several team meetings (see Table 1). The study's objectives guided the design of the questions, which focused on the emotional impact of subsequent waves of the pandemic, the nurses' experiences, and their reasons for intending to leave the profession, as well as the factors that influence their intention and decision.

The study participants were encouraged to share their experiences during the pandemic, including their feelings, thoughts, and personal reflections. Qualitative, descriptive studies normally use small samples [20]; however, the final sample size was determined through data saturation [21]. Data saturation is achieved when enough information has been obtained to replicate the study and when the ability to obtain additional information has been accomplished and further coding is no longer feasible [22].

### 3.1. Data Analysis

We performed thematic analysis following the method developed by Braun and Clarke [23] with assistance from the Atlas.tiV8 software. This analytical approach is employed in qualitative research to address general research inquiries. The outcome of thematic analysis consists of one or more themes that shed light on individuals' encounters, perspectives, and standpoints regarding the phenomenon in question [23]. The quotes were translated into English and validated by two bilingual members of the research team (JL and CW) to ensure that the translated quotes retained not only the syntax but also the original meaning. The data analysis process began with multiple readings, where a single researcher (JL) led the effort to acquaint himself with the data content. The researcher assigned descriptive codes to identified content pieces and subsequently categorized these codes according to similarities and differences. Following this, the researcher engaged in discussions over several sessions to reach a consensus with the rest of the research team and ensure the analysis's rigor and reliability. Through this iterative process, the data was comprehensively examined and interpreted from diverse perspectives, ensuring an accurate representation of participants' experiences.

### 3.2. Trustworthiness

We relied on the Consolidated Criteria for Reporting Qualitative Research (COREQ) checklist [24]. Throughout the research process, we adhered to the rigorous criteria proposed by Guba and Lincoln [25]. We closely and continuously monitored the process to ensure reliability, paying attention to the accuracy of the transcripts, and comparing them with the corresponding audio recordings. In addition, we carefully read the transcripts multiple times to guarantee the fidelity of the content. To ensure the accuracy and validity of the identified categories, two experts in qualitative methodology who were not part of the research team reviewed and confirmed the analytical process and results, which had also been approved by some participants. The research team members made efforts to identify any personal assumptions or preconceptions they may have held and to separate them from the study. The principal investigator was always available for debriefing and addressing any concerns or uncertainties during the process of analyzing the data.

### 3.3. Ethical Considerations

The study received ethical approval from the Ethics Committee for human and animal experimentation of the Autonomous University of Barcelona. Informed consent was obtained from all participants after providing them with detailed information about the study, including its importance and the procedures for maintaining confidentiality. Pseudonyms were used to ensure anonymity, and participants were assured that their information would be kept confidential, as expressed in the informed consent form. Additional measures were taken to protect participant privacy. All collected data were stored securely and only accessible to authorized members of the research team. Identifying information was kept separate from the main dataset to further preserve anonymity. During data analysis and reporting, aggregated findings were presented to prevent the identification of individual participants. Participants did not receive any financial incentives for their involvement in the study.

Ethical guidelines were strictly followed to ensure the protection of participants' rights and well-being throughout the study.

## 4. Results

### 4.1. Participant Characteristics

A total of 14 nurses, ranging in age from 27 to 61 years with an average of 22.8 years of work experience, participated in the study (see Table 2). The participant group included representation from all four Catalan provinces, with a majority of female participants. Moreover, the participants came from various professional backgrounds, which contributed to the study's broad range of perspectives.

### 4.2. Qualitative Results

All respondents reported considering leaving the profession at some point during the sixth and seventh waves of the COVID-19 pandemic. Thematic analysis of the data revealed three main themes: (1) the impact of COVID-19 on health; (2) factors influencing the decision to stay in the profession; and (3) recommendations to improve the management of similar situations (see Table 3).

### 4.3. The Impact of COVID-19 on Health

The participants reported feeling tired both physically and emotionally. The COVID-19 pandemic revealed the chronically precarious situation of the profession and the healthcare system in general. Nurses were exposed to exhaustive levels of pressure to care for patients as well as emotional fatigue that endangered their health and continuity in the field.

*I'm not sure if it was COVID-19 or because of COVID-19 that we realized how poorly we were doing. The arrival of COVID-19 made it clear to us. We had been at a low point for so long that I can't even recall how many years it's been. While COVID-19 is now under control, we remain at a low point, with exhausted, demotivated, and irritable people. The things we went through during the first waves [of the pandemic] are still present, and I have vivid memories of them. I've witnessed individuals pass away [before], but it wasn't simply the act of dying. It was the way in which it all unfolded—the disorder, the terror. To persevere through the initial and second instances was quite the feat, but these more recent waves have been something else entirely. Chaos continues to reign every single day. If—and when—another virus like COVID-19 arises, brace yourselves, because I truly don't think half of us could endure it. (P4)*

This reported lingering sense of professional uncertainty, combined with fatigue from the COVID-19 pandemic, led to a sense of disillusionment with the healthcare system, its management model, and nursing practice. Although all participants affirmed that the field has provided them with deep satisfaction, many of them indicated that nowadays, if given the choice, they would pursue a different career path because nursing has changed significantly and does not meet their expectations any longer.

*I would choose a different career, like art history or archaeology, if I could go back to high school. I wouldn't pick anything related to health. (P3)*

*I like being a nurse, but not this [kind of] nurse. (P8)*

*At work, I did notice the fatigue, and I also got a little burned out. I have come to question my work. (P5)*

After five previous waves of COVID-19, study participants faced the sixth and seventh waves with a significant toll taken regarding their physical and emotional health. Episodes of anxiety, depression, insomnia, irritability, and post-traumatic stress are particularly prominent at varying levels of severity. The symptoms of these conditions did not emerge in an acute and sudden manner but rather appeared due to long-standing professional dissatisfaction and cumulative fatigue.

*You know what's wrong? We are exhausted. It has been a challenging period, and it seems like everything has been forgotten. It's possible to lose motivation and passion as a result. The current daily routine exacerbates our situation since we are overworked and experiencing fatigue and impatience at the peak of our performance. I believe that sooner or later, some of us will eventually falter or give up and say, “You'll have to take it from here.” (P9)*

*I don't feel good. I'm tired and keep replaying our experiences repeatedly. I still can't sleep well, and it's been three years now. (P4)*

The participants needed professional assistance to manage the whirlwind of emotions that surfaced during the initial waves of the pandemic and persisted or worsened in subsequent waves. They acknowledged using the economic incentives provided by the government to cover expenses for sessions with physiotherapists, psychologists, or other professionals that were not covered by public health services.

*Since I graduated, I have consistently enjoyed going to work. I have learned a lot on the job and have always performed well. However, I have recently been feeling tired and disappointed, which has caused me anxiety for which I needed assistance. The extra pay they offered was not important to me. What I really needed was respect—recognition of what we endured and the support we required. In the end, having some cash was helpful to pay for the physical therapist and therapist. If not, many [nurses] would have probably [left]. I got a break from work due to my anxiety and I underwent private therapy, and now I am doing great! Moreover, I have been able to resume working, which is quite a lot. (P8)*

### 4.4. Factors Influencing the Decision to Stay in the Profession

During the first waves of the pandemic, the nurses did not contemplate abandoning the field. Despite their fear and fatigue, professional responsibility came before everything else. As one of the participants indicated, “*We could not give up at that time [in the first wave]. It was a very big responsibility”(P1).* However, when the epidemiological situation stabilized and there was no evidence of any change in the management of their precarious working conditions, in addition to a marked shortage of nurses, the participants began to take sick leave and had thoughts of abandoning the profession. The participants admitted that the pressure of the situation did not allow them to leave because they felt guilty. Quitting their jobs meant burdening their colleagues with more work since the possibility of finding replacements was practically nil. Therefore, on numerous occasions, they faced the dilemma of wanting to feel good themselves or sacrificing their well-being to make others feel good.

*In January 2021, I began taking medication for anxiety prescribed by my doctor so I could continue going to work. It was either that or take time off due to sickness, but I felt too unwell to do so. I experienced the common feeling of guilt because I believed my colleagues needed me, and I couldn't abandon them. (P12)*

However, the nurses acknowledged that to leave the field implies accepting defeat, and this is the chief reason why they remain in the profession, which they initially chose with enthusiasm and which has brought them happiness for many years despite the challenges. In addition, quitting nursing practice would mean losing the few labor rights and privileges they had obtained through their careers (particularly in terms of working hours, facility, and acquired seniority). The nurses were realistic and admitted that they have economic needs they are currently able to meet. They expressed uncertainty about their ability to meet those needs if they were to switch to another profession and start from scratch. As a result, despite their complaints and general dissatisfaction with the situation, they said they prefer to tolerate it rather than face financial instability or difficulties in terms of family reconciliation.

*I cannot give up. I still have many years of work ahead of me. It's my job, and I love it. I want more than just applause—I want real recognition. What I've gone through should serve a purpose beyond just keeping me going. (P1)*

*Then I considered the possibility of moving elsewhere and not being able to balance my work and family life. However, I decided against it because I currently have a schedule that allows me to be with my children and assist them as needed, even if I work on some weekends. If I move to another center, I may lose this routine and stability, which I prefer to maintain (P7).*

### 4.5. Recommendations for Improving the Management of Similar Situations

As for feeling professional fatigue and disillusionment, the nurses felt they were ignored during the pandemic, both at the political level and at the middle/higher levels in their workplaces. One of the participants referred to feeling “*ignored*” and “*belittled*.” Many of the participants expressed this view, revealing a pervasive sense of disappointment. The nurses acknowledged the exceptional nature of the situation but were highly critical of the management of access to protective materials, work shifts, breaks, and other measures, which were adopted without considering their input. As a result, the management of the situation adversely affected their physical and mental health, leading to dissatisfaction with the profession.

*Maybe they're not listening as much anymore. The mid-level managers seem busy, and it seems like the top executives are on their own. They believe we can handle everything, but we can't. Things used to be done differently. Things used to be done a certain way, but we were abruptly informed that they would now be done differently. They didn't directly ask us [for our input], as if we weren't important or weren't being considered. (P7)*

*We've been experiencing this throughout the pandemic. The authorities take care of themselves, and we have to tolerate whatever they decide, without any consideration for our opinions. We have to accept our situation and move forward. (P9)*

*I'm okay, but my voice trembles when you ask how I feel. We always say “fine” because there's no time to talk and we've been through too much to complain. But it upsets me and makes me angry that nobody cares how we really are. They applaud us but don't listen to us. (P8)*

The participants recognized the need to alter their approach to the profession, including their understanding, management, and even their attitude towards it. They realized the importance of adopting a model of emotional self-management centered on self-compassion and assertiveness over absolute dedication to others. This means placing the person and the professional in the foreground without affecting the quality of care, learning to listen to signs observed in the body, and knowing how to identify when it is necessary to pause without feeling guilty.

*We should prioritize carving out more leisure time to pursue personal growth and activities outside of the healthcare field. (P3)*

*I decided I needed a break and some rest. I [focused on] myself and didn't want to be all over the place anymore. I quit one job and now I have more time for myself. I do acupuncture and yoga to take care of myself. If we only focus on others, we'll spiral downward. Who takes care of you? (P9)*

The nurses invited the managers to become more involved with the emotional needs of their teams and to truly assess whether the care facility where they were located was the right one for them. They demanded that vacancies be filled, taking into account the experiences of the professionals as well as their personal preferences, thus facilitating a work-life balance.

*Nursing managers should consider their staff's opinions by asking them if they are content with their current location or if they would prefer a change. This simple gesture can improve employee satisfaction and motivation at work. (P2)*

## 5. Discussion

Our findings indicate that continuous exposure to COVID-19 impacted nurses' self-perceived health and well-being as well as their intention to leave the field. The participants expressed feelings of being ignored, enduring fatigue, and dealing with mental health challenges such as emotional instability, irritability, insomnia, and depression. Our results align with a growing body of evidence that highlights a surge in mental health problems among healthcare professionals during the COVID-19 pandemic [3, 26]. For instance, intensive care nurses experienced helplessness, exhaustion, and mental health issues; moreover, a significant percentage of primary healthcare nurses exhibited symptoms of depression, anxiety, and stress, which they attributed to the pandemic [27], and Martin-Rodriguez et al. [28] reported a significant prevalence of depression, anxiety, insomnia, and distress among nurses, particularly those working in COVID-19 units and nursing homes, who appeared to be particularly affected. Providing a broader context, a systematic review encompassing studies from 2020–2021 showed a high prevalence of moderate-to-severe symptoms of anxiety, depression, PTSD, and insomnia among nurses [3]. These findings collectively underscore the ubiquitous nature of mental health challenges among healthcare professionals during the COVID-19 pandemic.

The nurses felt abandoned by their healthcare institutions. They felt unsupported, especially during the early stages of the pandemic when uncertainty and fear were multiplying. The sense of abandonment expressed by our participants can be linked to the sense of being betrayed found in the literature discussing the concept of institutional betrayal among healthcare workers [29]. In this study, healthcare workers described frustration when their institutions did not prioritize their safety, and they believed that they had received inadequate compensation. Feelings of having been betrayed by their institutions were associated with increased burnout and a stronger intent to quit their jobs [29]. This highlights the critical need for healthcare organizations to foster a supportive and trustworthy environment for their staff, particularly during times of heightened uncertainty, such as during crises.

The present study indicates that nurses experienced significant changes in their working conditions, and they felt disillusioned at the professional level as well as personally transformed. These results align with prior research that has identified similar experiences of professional grief [30], turmoil, and personal transformation [31]. In essence, the present study suggests that nurses are deeply disappointed with the management system and with what the profession has become. Their profession is no longer recognized, which makes them think they would not choose nursing if they could go back in time and choose a different career. The findings underscore the disillusionment experienced by nurses with the current state of their profession, highlighting the urgent need for systemic changes to address their concerns and restore recognition and value to the nursing profession.

It is important to acknowledge the differences between our findings and those of prior research, where some nurses found renewed purpose and meaning in their roles during the pandemic [32, 33]. These differences may be attributed to the evolving nature of the pandemic, the varying experiences of healthcare professionals at different points in time, and our specific focus on individuals who have contemplated abandoning the profession. The differences in results underline the complexity of nurses' experiences during a crisis as well as the need for further research to better understand the shifting dynamics of nurses' professional lives. However, our findings partly align with those of Littzen-Brown et al. [32] as their participants described how the circumstances of the pandemic led them to provide suboptimal care. The consequences of this environment and the barriers to providing optimal nursing care had significant ramifications for the nurses, leading to constant feelings of frustration and being at a loss.

Finally, evidence suggests that a challenging work environment, a lack of support, emotional distress, and disappointment regarding the reality of the field were factors that reinforced nurses' motivation to leave the field [34]. This reality points to the urgent need for effective mechanisms to provide mental health support within healthcare organizations, as emphasized by our participants' suggestions for prioritizing personal well-being in future crisis management in order to foster retention.

We identified a number of factors that dissuaded nurses from abandoning the profession such as a sense of responsibility, feelings of guilt, pressure from management teams, economic considerations, and a desire to preserve working conditions. All these factors played pivotal roles in their decisions. In this regard, various factors influencing nurses' intention to stay or leave the field have been highlighted in the literature [9], including education level, mastery and expertise, perceived support, nurse-manager communication, stress levels, and family-related factors such as marital status or having children. These factors emphasize the individualized nature of nurses' decisions regarding their professional future. The interplay between individual motivations and organizational influences is essential in fully addressing nurses' intentions to remain in or leave the profession.

Several studies have examined the pandemic's impact on retention and turnover among nurses. In the present study, economic stability and favorable working conditions influenced nurses' decisions to stay in their roles. Squires [31] suggested that financial incentives, often linked to economic stability, are a driving force behind attrition rates. This highlights the undeniable connection between financial well-being and a nurse's decision to stay in the field, reinforcing the significance of economic considerations in nurses' career choices. It is important to note that while these factors resonated with our participants' experiences, there is also a degree of variability in how different nurses respond to these pressures. A study among senior nurses in Ireland showed that despite the adverse health impacts, some nurses responded positively to the pandemic, while others chose to retire early [35]. This variability demonstrates the multifaceted nature of nurses' decisions in the face of adversity.

Bahlman-van Ooijen's [34] qualitative systematic review indicates that nurses' reasons for quitting nursing practice include numerous difficulties, including a challenging work environment, emotional distress, disappointment about the reality of nursing, and a culture of hierarchy and discrimination. These findings offer valuable insights into areas where targeted interventions and improvements can be implemented. Furthermore, a longitudinal study conducted across two phases identified emotional states such as moral distress, compassion fatigue, burnout, and PTSD among nurses, which significantly influenced their thoughts of abandoning the profession [5]. The present study emphasizes the need to create a psychologically safe workplace to support retention among nurses. Additionally, frontline nurses experiencing compassion fatigue are at risk of lower job satisfaction and higher turnover intention [36].

In a broader context, the quality of the work environment significantly impacts nurses' risk of burnout and intention to leave the profession, with better work environments being associated with lower risks [37]. This aligns with our findings regarding the importance of preserving favorable working conditions to prevent job abandonment.

In this study, the nurses described the pivotal role of leadership, especially healthcare management, in nurturing the well-being of nurses during crises. Savage et al. [38] underscored the idea that healthcare leadership has the potential to function as both an asset and a barrier to organizational performance. Many participants described how leadership was unsure about how to provide structure and organization during the pandemic. Subsequently, nurses experienced a significant lack of support from leadership staff, such as their managers, who either were unavailable or left their positions. This observation is consistent with prior research highlighting the significance of effective leadership during challenging times [39]. Positive and supportive leadership positively affects nurses' commitment to the organization, leading to increased job satisfaction, productivity, retention, patient safety, and an overall safe climate [40]. Fowler and Robbins [41] emphasized the indispensable role of nursing leaders in enhancing efficiency, stressing the need for these leaders to exhibit both informational and motivational qualities, especially during healthcare challenges and crises. The participants' input demonstrates the critical need for strong, supportive leadership structures within healthcare organizations to mitigate the impact of crises on healthcare workers.

Moreover, the participants stressed the importance of personal growth and self-care as integral components of crisis management. This perspective resonates with the broader literature on resilience among nursing professionals, which emphasizes personal growth as a key element of their ability to navigate and overcome challenges [42]. The participants' insights suggest that the promotion of personal growth and self-care strategies should be integrated into crisis management plans to help healthcare professionals cope with the emotional challenges they may face. Practical aspects of crisis management were also underlined by nurses participating in the present study. Ball [43] found that nurses advocated for better personal protective equipment and support for the workforce. This pragmatic feedback indicates the importance of addressing tangible concerns to ensure the safety and well-being of healthcare workers during crises.

Finally, this study shows how nurses prioritize personal their well-being. Calkins [44], who identified feelings of inadequacy and exhaustion among intensive care nurses, further highlighted the urgent need for healthcare organizations to develop strategies to mitigate burnout and provide comprehensive support to their staff. The importance of peer support and an encouraging team culture in helping nurses cope with crises has also been emphasized in the literature [45], thus demonstrating the significance of fostering positive interpersonal relationships among healthcare professionals to enhance their resilience during challenging times. In line with this, Squellati and Zangaro [6] stressed the collaborative nature of coping with the demands of the healthcare profession, underlining the need for nurses to work together to support each other and their leaders in mitigating the situation and reducing burnout.

## 6. Limitations

This study has some limitations. The sampling method ensured the appropriateness of the participant profile in relation to the study's objectives. However, the sample is represented only by some nursing backgrounds and specific clinical settings. Moreover, the nurses participated voluntarily. Consequently, other potential options that could be explored in future studies were left out. This also means that the experiences of nurses who did not express interest in participating may have been overlooked due to challenges associated with their unease with the topic at hand. Nonetheless, the wide range of profiles and the repetition of data ensured their reliability and dependability. Additionally, the data align with the socioeconomic context of the specific context, precluding the possibility of extensive generalization. Nevertheless, we postulate that various outcomes could be applicable in similar populations of healthcare workers worldwide. Finally, there may have been some information bias due to the sensitive nature of the subject matter. Nevertheless, we took measures to reduce this and establish a safe environment of confidentiality and nonjudgment for the interviewees.

## 7. Future Research

For future research, conducting in-depth studies that focus on nurses who have left the profession could provide valuable insights into their experiences and reasons for departure. Understanding these factors in greater detail can inform targeted interventions and support strategies aimed at retaining healthcare professionals within the workforce.

Implementing action research methodologies could be beneficial in assessing the effectiveness of interventions designed to address nurses' concerns and improve retention rates. Action research principles, such as collaboration with stakeholders and iterative cycles of planning, action, and reflection, can actively involve nurses in the process of identifying and implementing solutions. This approach enables real-time adjustments based on feedback from participants, promoting a sense of ownership and engagement among healthcare professionals.

Additionally, taking into account the diverse backgrounds and experiences of nurses could improve the effectiveness of interventions. Customizing strategies to accommodate different needs and perspectives within the nursing workforce can result in more targeted and impactful solutions. By integrating insights from in-depth studies of nurses in this situation with action research methodologies, researchers can develop evidence-based strategies to address workforce challenges and enhance the sustainability of the nursing profession.

## 8. Conclusions

The emotional impact of the pandemic on the interviewed nurses was profound as they struggled with feelings of being overlooked, exhaustion, and mental health challenges. Despite the immense obstacles they faced, they demonstrated dedication to their field and professional commitment. Factors such as a sense of responsibility, guilt, and the importance of economic stability played pivotal roles in their decision to persevere. This resilience highlights the interconnections among personal motivation, organizational support, and economic stability in shaping career decisions during times of crisis.

There is an urgent need for comprehensive programs offering mental health support that are tailored to the unique demands of nursing. Recognizing and addressing emotional challenges must become a cornerstone of future crisis preparedness and response efforts. Apart from designing strategies to cope specifically with the effects of the COVID-19 pandemic on nurses, this study suggests the need for managers and decision-makers to propose sustainable actions that could overcome potentially similar situations. In addition, nurses' working conditions should be reviewed so that they can be retained and kept healthy, thereby avoiding situations of fatigue, stress, the intention to leave, and low motivation. Such circumstances have led to a worldwide nursing shortage and have hampered upward mobility to workplaces with better conditions.

Finally, policies and programs should be implemented at the management level that prioritize nurses' mental well-being and recognize their invaluable contributions. This multidimensional approach should include mental health support, leadership development, personal growth, and a culture of appreciation within healthcare organizations. By investing in nurses' well-being, we not only honor their dedication but also strengthen their resilience, ensuring that they can continue to deliver high-quality care during times of adversity.

## Figures and Tables

**Table 1:** Semistructured interview guide.

Semistructured interview guide
*Introduction*
Thank you for participating. Can you please start by telling me a bit about yourself and your background as a nurse?

*Understanding the impact of COVID-19*
How do you currently feel?
How would you describe your mental health today?
What relationship do you think it has with COVID or the pandemic we have experienced?
How has the COVID-19 pandemic affected your work as a nurse?
Can you describe any challenges or difficulties you have faced in your role during the pandemic?
How has the pandemic impacted your mental health and well-being?

*Exploring intention to leave the profession*
I see that you answered in the previous encounter that you have considered leaving the nursing profession, particularly during the COVID-19 pandemic, can you explain more?
What factors contributed to this consideration?
Can you describe any specific instances or experiences that led to thoughts of leaving the profession?
What factors ultimately influenced your decision to stay in or leave the nursing profession?

*Closing*
Is there anything else you would like to add or discuss regarding the impact of COVID-19 on your profession and mental health?
Do you have any final thoughts or reflections you would like to share before we conclude the interview?

**Table 2:** Sociodemographic data.

Variables	%
*Gender*	
Man	14
Woman	79
Other	7

*City*	
Barcelona	43
Tarragona	21
Lleida	21
Girona	14

*Professional setting*	
Hospital	43
Primary and community care	36
Correctional facility	14
Multiple settings	7

**Table 3:** Qualitative results (codes and themes).

Codes	Subtheme	Themes
They forgot about us	Feeling ignored	The impact of COVID-19 on health
Pressure from the management team		
Limited social recognition		
Lack of resources		
Primary invisibility		
Work overload	Feeling tired	
I'm overwhelmed		
Monotony		
COVID-19 was the last straw		
Professional demotivation		
Physical fatigue		
I'm feeling better, but it was horrible	Impact on mental health	
Emotional lability		
Irritability		
I'm getting by (idiom^1^)		
Insomnia		
Post-traumatic stress		
Depression		
Considering quitting for my health		
Taking medical leave		
Anxiety		
I wouldn't take up nursing again	Professional disappointment	
My job changed completely		
Feeling indecisive		
I need breaks		
Questioning my career in nursing (or questioning nursing itself)		

Responsibility	Factors influencing the decision to stay in the profession	
Feeling guilty		
Pressure from management teams		
For my financial situation		
Preserving working conditions		

The role of managers	Recommendations to improve the management of similar situations	
Nurturing personal growth		
Prioritizing personal well-being		

^1^Idiomatic expression means managing or handling the situation.

## Data Availability

The narrative data used to support the findings of this study have not been made available because of ethical reasons.

## Additional Points

*Statement of Clinical Relevance*. This study highlights the emotional impact of the COVID-19 pandemic on nurses and identifies critical factors that influence their intention to abandon the profession. Healthcare organizations can improve the well-being of their nursing staff, reduce turnover intentions, and ensure a resilient and dedicated workforce capable of delivering high-quality care, especially in times of crisis, by addressing these issues. Recognizing these challenges is crucial for healthcare administrators and policymakers to implement targeted strategies for improving working conditions. Prioritizing mental well-being policies and fostering a culture of appreciation are essential for sustaining nursing excellence during adversity.
